# Disease-free survival as a surrogate endpoint for overall survival in adjuvant trials of pancreatic cancer: a meta-analysis of 20 randomized controlled trials

**DOI:** 10.1186/s12885-020-06910-5

**Published:** 2020-05-14

**Authors:** Run-Cong Nie, Xue-Bin Zou, Shu-Qiang Yuan, Ying-Bo Chen, Shi Chen, Yong-Ming Chen, Guo-Ming Chen, Xiao-Jiang Chen, Tian-Qi Luo, Shu-Man Li, Jin-Ling Duan, Yun Wang, Yuan-Fang Li

**Affiliations:** 1 Department of Gastric Surgery, Sun Yat-sen University Cancer Center, State Key Laboratory of Oncology in South China, Collaborative Innovation Center for Cancer Medicine, Guangzhou, China; 2 Department of Ultrasound, Sun Yat-sen University Cancer Center, State Key Laboratory of Oncology in South China, Collaborative Innovation Center for Cancer Medicine, Guangzhou, China; 3 Department of Gastric Surgery, The 6th Affiliated Hospital, Sun Yat-sen University, Guangzhou, China; 4 Department of Experimental Research (Cancer Institute), Sun Yat-sen University Cancer Center, State Key Laboratory of Oncology in South China, Collaborative Innovation Center for Cancer Medicine, Guangzhou, China; 5 Department of Hematologic Oncology, Sun Yat-sen University Cancer Center, State Key Laboratory of Oncology in South China, Collaborative Innovation Center for Cancer Medicine, No. 651 Dongfeng Eastern Road, Guangzhou, 510060 Guangdong China

**Keywords:** Pancreatic cancer, Disease-free survival, Overall survival, Surrogate

## Abstract

**Background:**

We aimed to assess whether disease-free survival (DFS) could serve as a reliable surrogate endpoint for overall survival (OS) in adjuvant trials of pancreatic cancer.

**Methods:**

We systematically reviewed adjuvant randomized trials for non-metastatic pancreatic cancer after curative resection that reported a hazard ratio (HR) for DFS and OS. We assessed the correlation between treatment effect (HR) on DFS and OS, weighted by sample size or precision of hazard ratio estimate, assuming fixed and random effects, and calculated the surrogate threshold effect (STE). We also performed sensitivity analyses and a leave-one-out cross validation approach to evaluate the robustness of our findings.

**Results:**

After screening 450 relevant articles, we identified a total of 20 qualifying trails comprising 5170 patients for quantitative analysis. We noted a strong correlation between the treatment effects for DFS and OS, with coefficient of determination of 0.82 in the random effect model, 0.82 in the fixed effect model, and 0.80 in the sample size weighting; the robustness of this finding was further verified by the leave-one-out cross-validation approach. Sensitivity analyses with restriction to phase 3 trials, large trials, trials with mature follow-up periods, and trials with adjuvant therapy versus adjuvant therapy strengthened the correlation (0.75 to 0.88) between DFS and OS. The STE was 0.96 for DFS.

**Conclusions:**

Therefore, DFS could be regarded as a surrogate endpoint for OS in adjuvant trials of pancreatic cancer. In future similar adjuvant trials, a hazard ratio for DFS of 0.96 or less would predict a treatment impact on OS.

## Background

Pancreatic cancer is one of the few malignant tumors with increasing incidence and mortality in both sexes [1], and it is predicted to become the third leading cause of death in the European Union in 2020 [2]. Fewer than 20% of pancreatic cancer patients present at a localized, resectable stage at their first visit, and curative resection remains the only chance of cure for these patients. Progress in surgical techniques in recent years has likely minimized postoperative complications, which is regarded as an important factor in long-term survival [3, 4]. However, in the absence of adjuvant therapy, approximately 90% of patients suffered from distant or local relapse within 5 years after curative resection, and curative resection alone only yields a 5-year overall survival (OS) of approximately 8 to 13% [5–7]. Thus, valid adjuvant therapies are required to reduce this risk.

Several effective therapeutic strategies have been demonstrated to be effective for resectable pancreatic cancer [5–12], among which adjuvant chemotherapy can significantly reduce the risk of relapse and improve the survival of pancreatic cancer after curative resection [5–10]. To date, adjuvant gemcitabine and S^− 1^ remains the first recommendation for non-Asian and Asian patients after resection, respectively. However, the objective response rate of single-agent chemotherapy in the metastatic stage was reported to be low, in the range of 7 to 21% [13–15]. The landmark CONKO-001 (Charité Onkologie 001) study showed that 133 of 179 patients (74.3%) suffered from local relapse (25.3%) or distant metastasis (49.0%) after adjuvant gemcitabine treatment [16]. Therefore, clinicians are exploring whether more intensive therapeutic strategies, including combination regimens [17–19], adjuvant chemoradiotherapy [5, 10, 20, 21] and adjuvant immunotherapies [22–24], could enhance the therapeutic efficacy and translate to a survival benefit. For example, the PRODIGE 24/CCTG PA.6 trial further demonstrated that modified FOLFIRINOX regimen could lead to statistically prolonged RFS and OS than gemcitabine for patients with resected pancreatic cancer [19].

The gold standard endpoint in adjuvant trials of pancreatic cancer is OS, which has the advantage of being simple and reliable to measure, straightforward to interpret, and clinically useful. However, this endpoint has its disadvantages: it requires many patients and lengthy follow-up duration to detect statistically significant differences. In addition, its estimates are potentially diluted by non-cancer deaths and subsequent therapies after recurrence. Therefore, reliable endpoints that could be used as surrogates for OS in pancreatic cancer could shorten the follow-up period and reduce the cost of drug development. Among them, disease-free survival (DFS) is the reasonable potential surrogate endpoint for OS in the adjuvant setting of pancreatic cancer. Several meta-analyses have revealed that DFS is validated as a surrogate for OS in lung cancer [25], gastric cancer [26] and colorectal cancer [27]. Although Petrelli et al. reported that DFS cannot represent a reliable surrogate endpoint for OS in adjuvant trials of pancreatic cancer [28], the number of included trials in that study was comparatively small (12 trials); additionally, among the 12 trials, one was the adjuvant trial of periampullary adenocarcinoma (the ESPAC-3 periampullary cancer randomized trial) rather than pancreatic cancer [29], which would confound the results.

Therefore, with the accumulated evidence of 20 randomized controlled trials, we performed a rigid meta-analysis to evaluate whether DFS could be used as a surrogate endpoint to measure the effect of the adjuvant therapy of pancreatic cancer.

## Methods

### Search strategy and data collection

In December 2018, we searched Medline and Embase systematically using the key words “pancreatic neoplasm”, “chemotherapy”, “radiotherapy”, and “chemoradiotherapy”, limited to “clinical trial”, “controlled clinical trial” or “randomized controlled trial”. We also search the ClinicalTrials. Gov and Cochrane Library databases, and manually searched the references of the included trials and abstracts of two conference proceedings (the 2019 American Society of Clinical Oncology [ASCO] annual meeting and the European Society for Medical Oncology [ESMO] 2018 congress) to retrieve additional studies.

Inclusion criteria were randomized controlled trials of adjuvant treatment for non-metastatic pancreatic cancer after curative resection, reporting hazard ratio (HR) for OS and DFS in full-text publication. We excluded reviews, abstracts, case reports, studies that were not published as full-text articles and studies with cohorts of less than 50 patients. For each trial, the following data were collected by two independent investigators (RCN and SQY): OS and DFS results, final publication year, trial conduct period, type of study (phase II or III), staging information, treatment arms, number of patients, primary endpoint, and median follow-up time.

### Statistical analysis

This analysis is at the trial level throughout, with no individual patient-level data being incorporated. We computed the correlation between the treatment effect (HR) on DFS and OS through a linear regression model [27]. To interpret the differences between studies regarding study size and precision of HR estimates, we weighted the analysis proportionally to the study sample size or to the precision of the observed treatment effects. Hence, we applied three weighting strategies (sample size, fixed effect, and random effect) as the weighting strategies [30]. While the fixed effect meta-analysis is based on the presumption that a common treatment effect exists among every trial and uses the estimated inverse variance as weights, the random effect meta-analysis permits treatment effect discrepancy from trial to trial and merges the potential among-trial variation of effects into the weights. According to A’ Hern et al. [31], we down-weighted the sample size if trials reported more than two treatment arms.

We calculated the weighted coefficient of determination (*R*^*2*^) to quantify the variation explained by the surrogate endpoints, with *R*^*2*^ value higher than 0.75 as a strong correlation, higher than 0.5 as good, higher than 0.25 as moderate, and equal to or lower than 0.25 as poor. We performed several sensitivity analyses that restricted the analyses to phase 3 trials, large trials (included patients ≥200), trials with mature follow-up periods (median follow-up ≥24 months), trials with adjuvant therapy versus observation, and trials with adjuvant therapy versus adjuvant therapy to verify the robustness of our findings. We also calculated the surrogate threshold effect (STE), which was defined as the minimum treatment effect on the surrogate necessary to predict an OS benefit [32]. The upper limit of the confidence interval for the estimated surrogate treatment effect should fall below the STE to predict a non-zero effect on OS. For each meta-analysis, we applied an internal validation through leave-one-out analysis to evaluate the prediction accuracy of the surrogate model [33]. Each trial was left out once, and the surrogate model was built with other trials. This model was then re-applied to the left-out trial, and a 95% prediction interval was calculated to compare the predicted and observed treatment effect on OS. We used R version 3.4.0 for all statistical analyses (http://www.r-project.org).

## Results

**Fig. 1:**
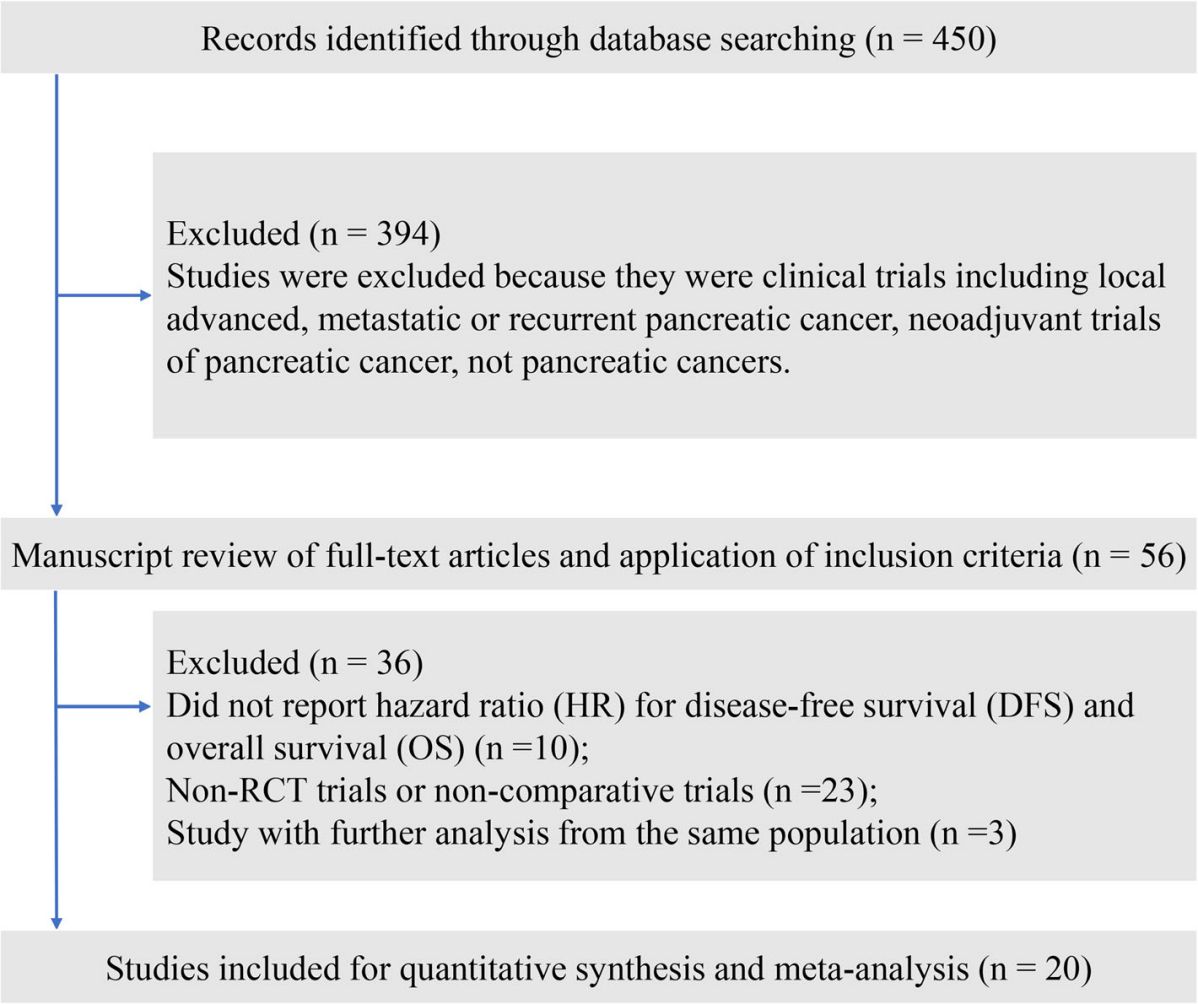
Study flow diagram of the included studies in this meta-analysis

After the systematic literature review, we identified 20 qualifying trials (5 phase 2 trials and 15 phase 3 trials) comprising 5170 patients for final analysis (Fig. 1, Table 1) [5–10, 17–24, 34–39]. The median follow-up period of the included trials varied from 17.0 months to 104.4 months. The ESPAC-1 trial (European Study Group for Pancreatic Cancer-1) [10] was designed as a two-by-two factorial design to evaluate the role of adjuvant chemoradiotherapy and chemotherapy independently, with 75 patients randomly divided into the chemotherapy group, 73 patients in the chemoradiotherapy group, 72 patients in the chemoradiotherapy and chemotherapy group, and 69 patients in the observation group. Neoptolemos et al. reported the interim result of ESPAC-1 trial in 2001 [40], and updated the long-term survival outcomes after a median follow-up of 47.0 months [10]; thus, we included the latter publication in the present study. The CONKO-001 trial was also first published in 2007 [16] and was updated in 2013 [7]. Overall, the 20 trials included 23 comparisons for quantitative analysis, among which nine comparisons reported improvement in OS, and eleven comparisons reported improvement in DFS **(**Table 2**)**.

**Table 1:** Characteristics of the included studies

Studies	Final pub year	Trial conduct period	Type of study	Stage	Treatment arms	Number of patients	Primary endpoint	Median follow-up (months)
Kalser et al. [5]	1985	1974–1982	III	R0	CRT vs. observation	43	OS	66
Lygidakis et al. [8]	2002	1993–2000	III	Stage III	CIT vs. AC vs. observation	128	OS	NR
Takada et al. [9]	2002	1986–1992	III	Stage II-III	AC vs. observation	158	OS	60.0
Neoptolemos et al. [10]^a^	2004	1994–2000	III	R0/1	CRT vs. AC vs. observation	289	2-year OS rate	47.0
Kosuge et al. [34]	2006	1992–2000	III	R0	AC vs. observation	89	OS	44.8
Smeenk et al. [20]	2007	1987–1995	III	T_1-3_N_0–1a_M_0_	CRT vs. observation	218	OS	140.4
Morak et al. [35]	2008	2000–2007	III	Stage I-III	CAI/RT vs. observation	120	OS	17.0
Yoshitomi et al. [17]	2008	2002–2005	II	R0/1	AC vs. AC	99	DFS	21.0
Ueno et al. [6]	2009	2002–2005	III	R0/1	AC vs. observation	118	OS	60.4^b^
Neoptolemos et al. [36]	2010	2000–2007	III	R0/1	AC vs. AC	1088	OS	34.2^b^
Van Laethem et al. [21]	2010	2004–1007	II	R0	CRT vs. AC	90	Treatment completion	33.3
Schmidt et al .[22]^c^	2012	2004–2007	III	R0/1	CRT + IFN -2b vs. CRT/AC	110	OS	45.9
Oettle et al .[7]^d^	2013	1998–2004	III	T_1-4_N_0–1_ M_0_	AC vs. observation	384	DFS	136
Shimoda et al. [37]	2015	2008–2012	II	R0/1	AC vs. AC	57	DFS	NR
Uesaka et al .[38]^c^	2016	2007–2010	III	Stage I-III	AC vs. AC	377	OS	82.3
Neoptolemos et al. [39]	2017	2008–2014	III	R0/1	AC vs. AC	730	OS	43.2
Sinn et al. [18]	2017	2008–2013	III	R0	AC vs. AC	436	DFS	54.0
Reni et al. [24]	2018	2006–2008	II	R0/1	CRT vs. CRT	130	Toxicity	NR
Berlin et al. [23]	2018	2010–2015	II	Stage I–II	AC vs. AC	56	DFS	55.4
Conroy et al. [19]	2018	2012–2016	III	R0/1	AC vs. AC	493	DFS	33.6

*OS* overall survival, *DFS* disease-free survival, *CRT* chemoradiotherapy, *AC* adjuvant chemotherapy, *RT* radiation therapy, *CIT* chemoimmunotherapy, *CAI* celiac artery infusion, *NR* not reported

^a^This trial was designed as a two-by-two factorial design to test two comparisons: chemoradiotherapy, and chemotherapy. Patients were randomly assigned to chemoradiotherapy-alone group (*n* = 73), chemotherapy-alone group (*n* = 75), both chemoradiotherapy and chemotherapy group (*n* = 72), and observation group (*n* = 69)

^b^Follow-up for the living patients

^c^These trials were analyzed by per-protocol population

^d^The long-term outcomes of CONKO-001 trial

**Table 2:** Disease-free survival and overall survival estimate for the included trials

Study	Number of patients		Disease-free survival		Overall survival	
	Experimental arm	Control arm	Hazard ratio	95% CI	Hazard ratio	95% CI
Kalser et al. [5]	21	22	0.45	0.25–0.83	0.51	0.28–0.94
Lygidakis et al. [8]						
CIT vs. AC	43	45	0.63	0.42–0.96	0.61	0.40–0.93
CIT vs. observation	43	40	0.49	0.32–0.75	0.60	0.39–0.92
AC vs. observation	45	40	0.57	0.37–0.87	0.65	0.42–1.00
Takada et al. [9]	81	77	0.97^a^	0.93–1.30	0.86	0.63–1.18
Neoptolemos et al .[10]^b^						
CRT vs. no CRT	145	144	1.27	1.01–1.60	1.28	0.99–1.66
AC vs. no AC	147	142	0.76	0.60–0.96	0.71	0.55–0.92
Kosuge et al. [34]	45	44	1.03	0.68–1.56	1.18	0.78–1.79
Smeenk et al. [20]	110	108	0.94	0.70–1.26	0.91	0.68–1.23
Morak et al. [35]	59	61	0.64	0.45–0.92	0.81	0.57–1.16
Yoshitomi et al. [17]	50	49	1.09	0.74–1.62	1.24	0.84–1.84
Ueno et al. [6]	58	60	0.60	0.40–0.89	0.77	0.51–1.14
Neoptolemos et al. [36]	537	551	0.96	0.84–1.10	0.94	0.81–1.08
Van Laethem et al. [21]	45	45	1.00	0.66–1.51	1.01	0.67–1.53
Schmidt et al. [22]^c^	53	57	0.91	0.63–1.31	0.88	0.61–1.27
Oettle et al. [7]^d^	179	175	0.55	0.44–0.69	0.76	0.61–0.95
Shimoda et al. [37]	29	28	0.67	0.40–1.11	0.70	0.36–1.36
Uesaka et al. [38]^c^	187	190	0.60	0.47–0.76	0.57	0.44–0.72
Neoptolemos et al. [39]	364	366	0.86	0.73–1.02	0.82	0.68–0.98
Sinn et al. [18]	219	217	0.94	0.76–1.15	0.93	0.70–1.23
Reni et al. [24]	67	63	1.12	0.78–1.61	1.06	0.73–1.55
Berlin et al. [23]	30	26	0.53	0.30–0.96	0.86	0.41–1.81
Conroy et al. [19]	247	246	0.58	0.46–0.73	0.64	0.48–0.86

^a^Hazard ratio for 5-year disease-free survival

^b^This trial was designed as a two-by-two factorial design to test two comparisons: chemoradiotherapy, and chemotherapy. Patients were randomly assigned to chemoradiotherapy-alone group (*n* = 73), chemotherapy-alone group (*n* = 75), both chemoradiotherapy and chemotherapy group (*n* = 72), and observation group (*n* = 69)

^c^These trials were analyzed by per-protocol population

^d^The long-term outcomes of CONKO-001 trial

**Fig. 2:**
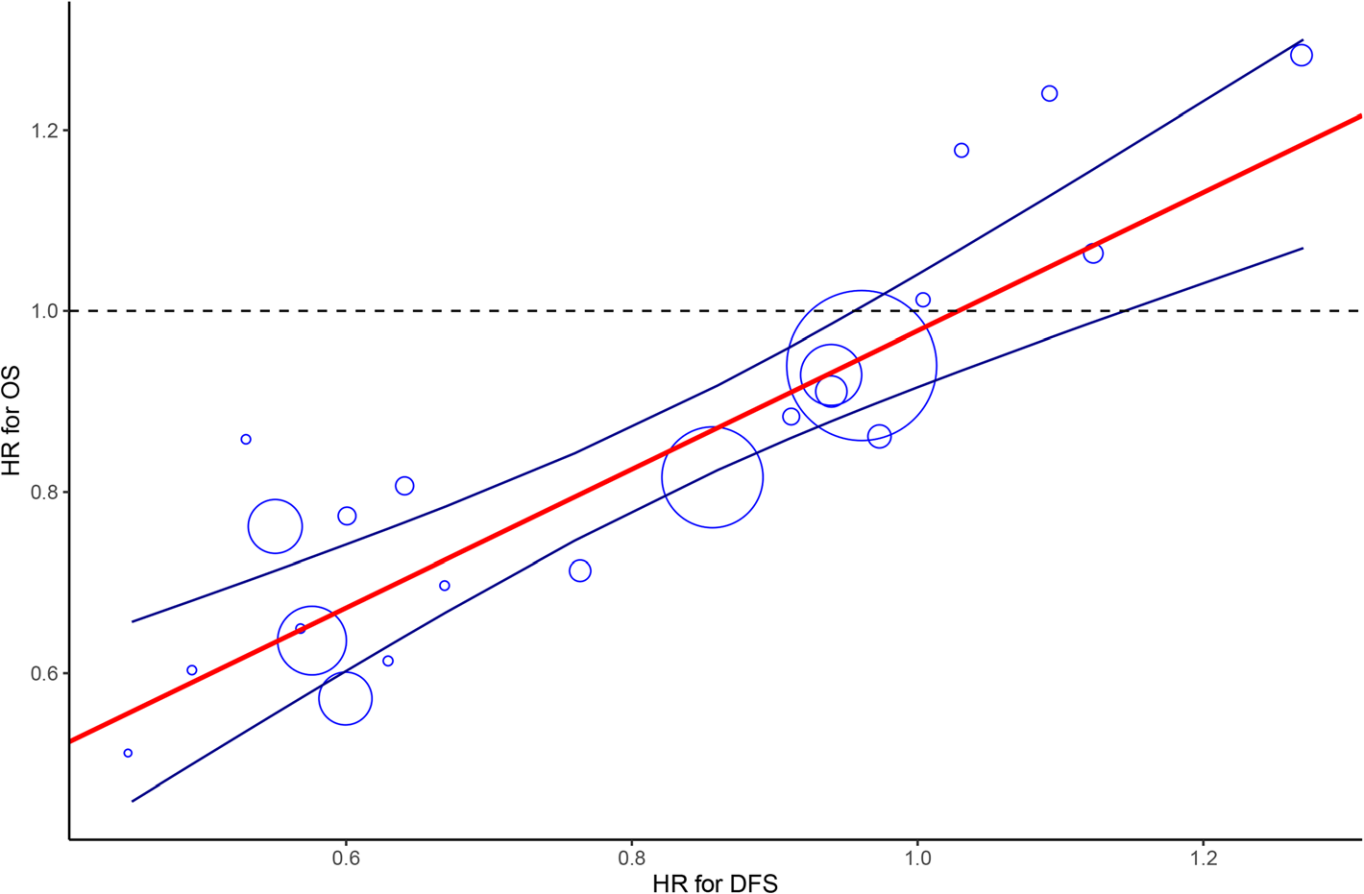
Correlation between treatment effects on DFS and OS. Each trial is represented by a circle, with the size of the circle being proportional to the sample size. The blue line represents the 95% prediction limit of the regression line (red line). STE = 0.96; OS, overall survival; DFS, disease-free survival; STE, surrogate threshold effect; HR, hazard ratio

We first assessed the degree of association through sample size weighting strategy, and observed that the correlation between the treatment effect on DFS and OS was strong (*R*^2^ = 0.80, 95% CI: 0.49 to 0.99) (Fig. 2). Additionally, we noted that permitting difference (random effect model) and no difference (fixed effect model) between therapy type and treatment effect on DFS and OS slightly strengthened the degree of association (fixed effect: 0.82, 0.52 to 0.99; random effect: 0.82, 0.52 to 0.99). We then calculated the STE of 0.96, indicating that a future adjuvant trial would need less than 0.96 for DFS of the upper limit of the confidence interval to predict with 95% confidence an OS benefit.

**Fig. 3:**
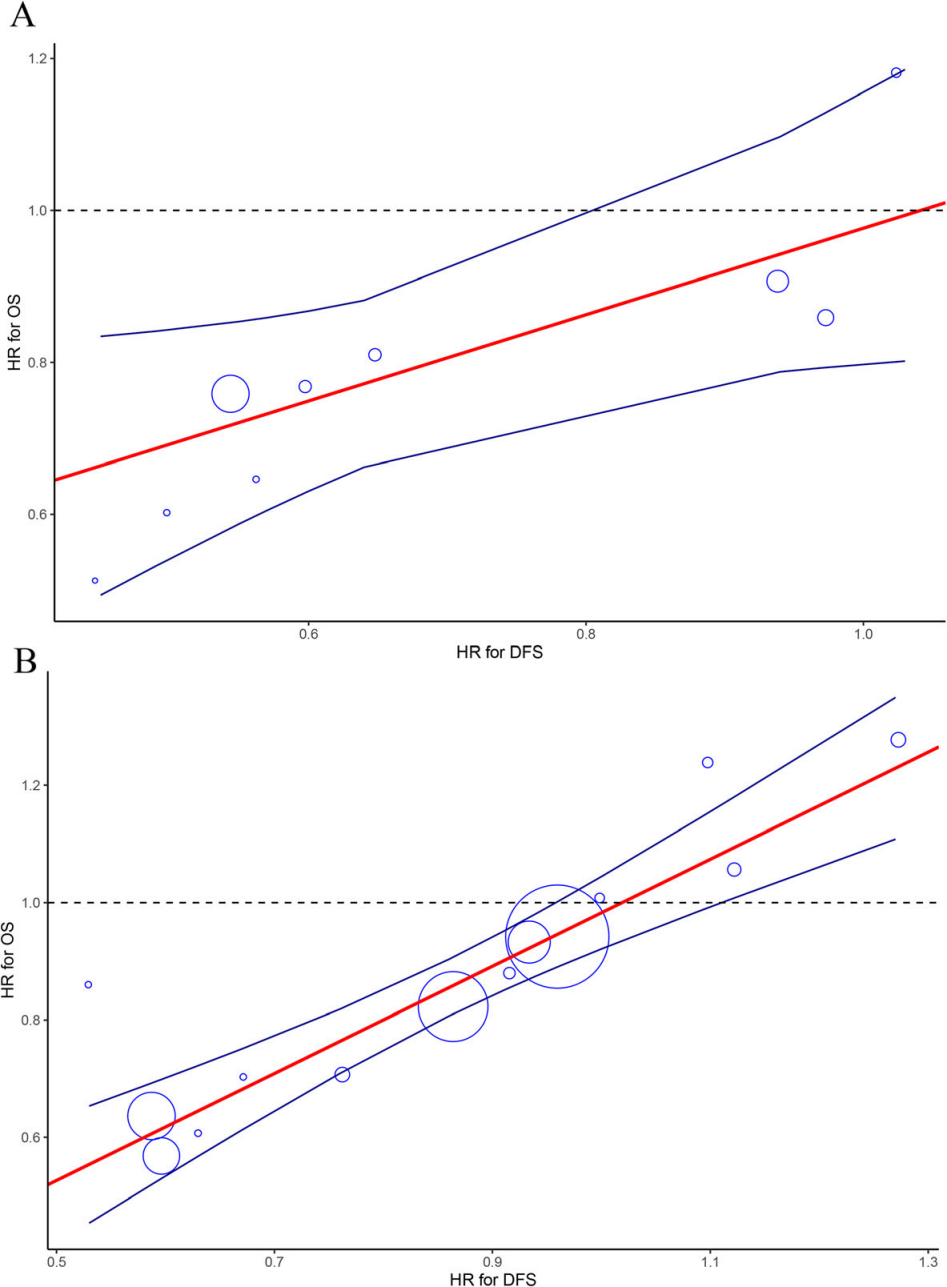
Correlation between treatment effects on DFS and OS (related to Table 3) according the sensitivity analysis that restricted to trials with adjuvant therapy versus observation (**a**) and trials with adjuvant therapy versus adjuvant therapy (**b**). Each trial is represented by a circle, with the size of the circle being proportional to the sample size. The blue line represents the 95% prediction limit of the regression line (red line). OS, overall survival; DFS, disease-free survival; HR, hazard ratio

Given the potential heterogeneity of the included studies, we performed several sensitivity analyses (Table 3), and noted that restriction of the analysis to phase 3 trials would strengthen the correlation between DFS and OS (0.82 to 0.83). When we restricted the analyses to trials with adjuvant therapy versus observation, the degree of association between DFS and OS was not strong (0.68 to 0.73) (Fig. 3a). Nonetheless, we recognized that adjuvant therapy versus adjuvant therapy rather than observation is now the standard design setting for pancreatic cancer; thus, we then restricted the analyses to trials with adjuvant therapy versus adjuvant therapy, and observed a very strong correlation between DFS and OS (0.89 to 0.93). Other sensitivity analyses that restricted the analyses to large trials and trials with mature follow-up periods also exhibited strong correlations between DFS and OS (0.80 to 0.87) (Fig. 3b).

**Table 3:** Sensitivity analysis

	*R*^2^ (95% CI)	*P* value	STE
**Total population** [5–10, 17–24, 34–39]			**0.96**
Sample size	0.80 (0.49 to 0.99)	< 0.001	
Fixed effect	0.82 (0.52 to 0.99)	< 0.001	
Random effect	0.82 (0.52 to 0.99)	< 0.001	
**Phase 3 trials** [5–10, 18–20, 22, 34–36, 38, 39]			**0.96**
Sample size	0.82 (0.48 to 0.99)	< 0.001	
Fixed effect	0.82 (0.49 to 0.99)	< 0.001	
Random effect	0.83 (0.50 to 0.99)	< 0.001	
**Trials with overall included patients ≥ 200** [7, 10, 18–20, 36, 38, 39]			**0.93**
Sample size	0.85 (0.41 to 0.99)	< 0.001	
Fixed effect	0.86 (0.41 to 0.99)	< 0.001	
Random effect	0.87 (0.44 to 0.99)	< 0.001	
**Trials with median follow-up ≥ 24 months** [6, 7, 9, 10, 18–23, 34, 36, 38, 39]			**0.95**
Sample size	0.80 (0.43 to 0.99)	< 0.001	
Fixed effect	0.81 (0.45 to 0.99)	< 0.001	
Random effect	0.80 (0.43 to 0.99)	< 0.001	
**Trials with adjuvant therapy versus observation** [5–9, 20, 34, 35]			**0.81**
Sample size	0.68 (0.17 to 0.99)	0.006	
Fixed effect	0.69 (0.18 to 0.99)	0.005	
Random effect	0.73 (0.22 to 0.99)	0.003	
**Trials with adjuvant therapy versus adjuvant therapy** [8, 17–19, 21–24, 36, 38, 39]			**0.96**
Sample size	0.90 (0.59 to 0.99)	< 0.001	
Fixed effect	0.93 (0.66 to 0.99)	< 0.001	
Random effect	0.89 (0.58 to 0.99)	< 0.001	

*R*^2^ coefficient of determination, *STE* surrogate threshold effect

**Fig. 4:**
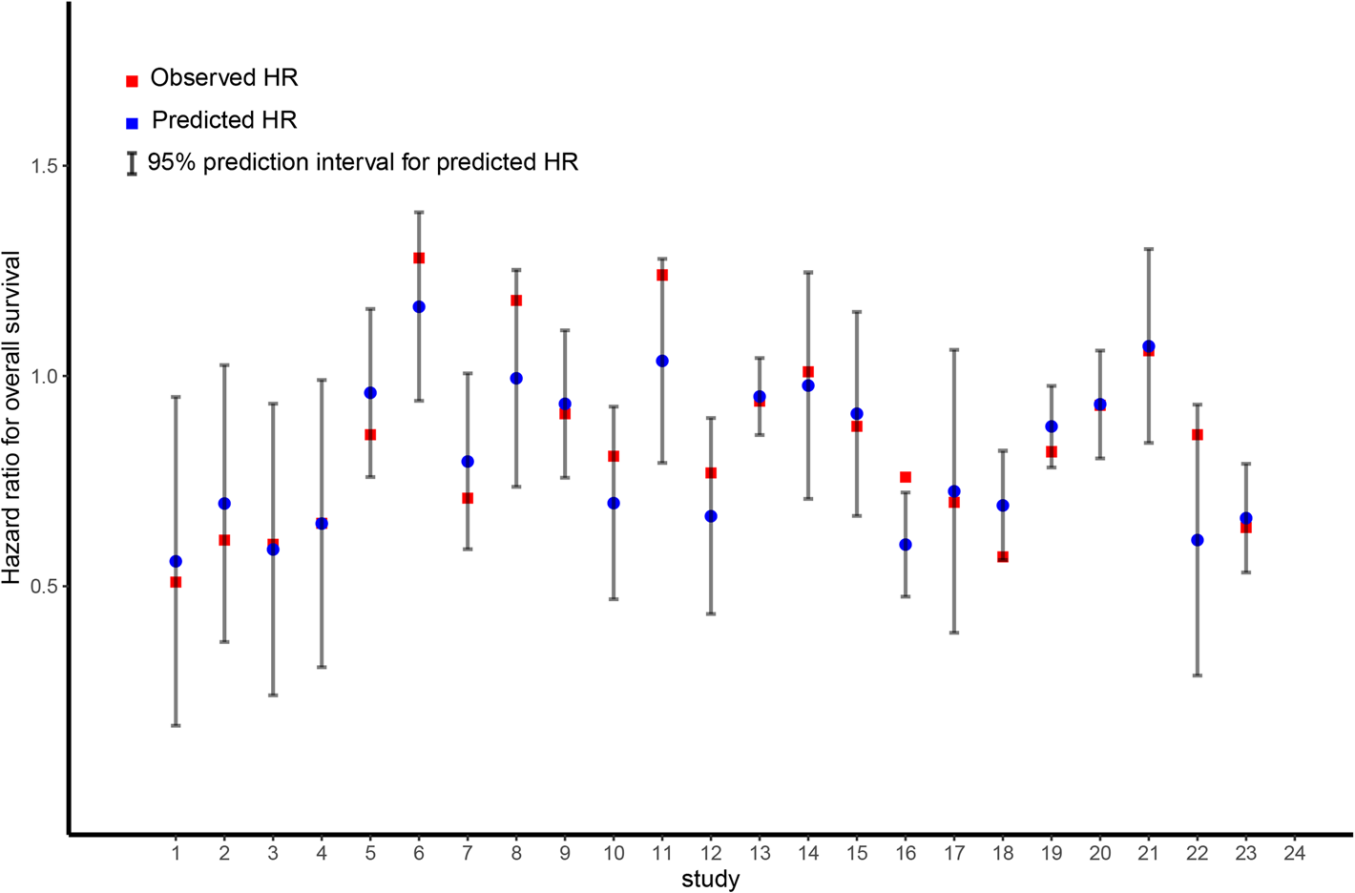
Leave-one-out cross-validation analysis of the prediction of OS by treatment effect on DFS: observed HR for OS for left-out trial vs. predicted HR for OS and 95% prediction interval for predicted HR for OS. To assess model accuracy, a leave-one-out cross-validation strategy was used: each unit of analysis was left out once, and the linear model was then constructed from scratch using the remaining data [33]. This model was then re-applied to the left-out study in order to compare the predicted and observed treatment effect on OS. Based on the linear regression models, a 95% prediction interval was calculated compare the predicted and observed treatment effect on OS. OS, overall survival; DFS, disease-free survival; HR, hazard ratio

Finally, we performed a leave-one-out cross validation approach to assess the accuracy of DFS in predicting OS. We noted that the observed HR for OS fell between the limits of the 95% prediction intervals in 22 of 23 comparisons, indicating that the treatment effect on DFS is a reliable predictor of OS (Fig. 4).

## Discussion

The point at which a potential surrogate endpoint could be theoretically validated has been seriously discussed [41]. The correlation approach has been widely adopted to validate the efficiency of a surrogate endpoint in locally advanced lung cancer [25], gastric cancer [26, 42] and colorectal cancer [27]. In the present study, we included a total of 20 high quality adjuvant randomized controlled trials to evaluate the surrogacy of DFS for OS in pancreatic cancer. Our finding demonstrated that the correlation between DFS and OS was strong (0.80 to 0.82), irrespective of the applied weighting strategies. Sensitivity analyses that were restricted to phase 3 trials, large trials, trials with mature follow-up periods, and trials with adjuvant therapy versus adjuvant therapy also yielded strong or very strong correlations (0.80 to 0.93) between DFS and OS. Therefore, we proposed the use of DFS as the surrogate endpoint for OS in adjuvant trials of pancreatic cancer.

Although the recent advance in adjuvant chemotherapy have translated into substantial survival benefit for pancreatic cancer, a large number of these treated patients still suffered from relapse or metastasis; thus, new therapeutic strategies are urgently needed. Clinicians are now evaluating the therapeutic effect of more intensive adjuvant chemotherapy, adjuvant targeted therapy and immunotherapy in pancreatic cancer after curative resection. It is well recognized that OS is the standard endpoint for clinical trials; however, using the endpoint of OS to perform the phase 3 trials is time consuming, thus postponing the new therapy strategies in clinical application. Therefore, we urgently need reliable surrogate endpoints for OS in adjuvant trials of pancreatic cancer, among which DFS is the most reasonable surrogate endpoint, and it has been set as the primary endpoint in several phase 3 trials [7, 17–19, 23, 37]. A previous meta-analysis reported that the correlation between DFS and OS was not strong enough to support the DFS as the reliable surrogate endpoint for OS in adjuvant trials of pancreatic cancer [28]; nonetheless, they only included a total of 12 trials, among which one trial was adjuvant setting for periampullary cancer rather than pancreatic cancer [29]. Therefore, in the present meta-analysis, we applied more rigorous criteria through three weighting strategies to address this urgent issue. Our findings revealed that the degree of association between DFS and OS was strong, which was further verified through extensive sensitivity analyses and a leave-one-out analysis validation approach. We believe that the robust correlation between DFS and OS in adjuvant therapy of pancreatic cancer is mainly attributable to the fact that pancreatic cancer is an aggressive tumor and that the subsequent lines of therapy are limited if patients develop relapse or metastasis.

Given the fact that adjuvant chemotherapy has showed superior survival outcome to observation for pancreatic cancer, adjuvant chemotherapy including gemcitabine-based or S-1-based regimens rather than observation would be set as the control arm in adjuvant trials. Interesting, we found that the correlation between DFS and OS was not strong (0.68 to 0.73) with restriction to trials with adjuvant therapy versus observation; nonetheless, we noted a very strong correlation between DFS and OS when we restricted the analysis to trials with adjuvant therapy versus adjuvant therapy (0.89 to 0.93). Therefore, in future adjuvant trials of pancreatic cancer, DFS could be served as the robust surrogate endpoint for OS.

STE is an alternative measure for surrogate endpoint validation [32]. Using a surrogate endpoint with STE closer to 1, it would be easier to predict an OS benefit. In the present meta-analysis, our finding showed that the STE was 0.96 for DFS, indicating that an adjuvant trial in pancreatic cancer producing a hazard reduction of at least 4% for disease recurrence or death could be expected to promise a statistically significant reduction in OS.

There are several limitations that should be noted. First, the data for our analysis were extracted from trial level rather than an individual patient; therefore, a potential published bias cannot be excluded. Second, the included trials spanned nearly three decades, and the ascertainment of DFS was mainly influenced by the image examination and surveillance interval, thus may have changed considerably over time and among trials. Third, long-term follow-up was not available from all trials included in our analysis. Pancreatic cancer is a relatively aggressive malignancy with severe heterogeneity; thus, the short follow-up in adjuvant trials will result in fairly wide confidence intervals of HR about the treatment effects. In the sensitivity analysis, the correlation between DFS and OS remained strong (*R*^2^ = 0.75) when we included trials with median follow-up > 24 months. Third, the included trials at our analysis comprised a wide range of therapeutic strategies, which included trials of adjuvant chemotherapy, radiation therapy, chemoradiotherapy, chemoimmunotherapy and targeted treatment. Although we performed sensitivity analysis to eliminate the potential effect of these treatment heterogeneities, the results of our analysis should be interpreted with caution. Therefore, we strongly recommended authors of individual trials to share their data to further verify the results of our analysis through individual-patient data.

## Conclusions

In conclusion, our analysis suggested that DFS could serve as a reliable surrogate endpoint for OS in adjuvant trials of pancreatic cancer. In future similar adjuvant trials, a hazard ratio for DFS of 0.96 or less would predict a treatment impact on OS. However, these results should be further verified by individual-patient data analysis.

## Data Availability

All data generated or analysed during this study are included in this published article.

## Abbreviations

OS: Overall survival
DFS: Disease-free survival
ASCO: American Society of Clinical Oncology
ESMO: European Society for Medical Oncology
HR: Hazard ratio
R2: Coefficient of determination
STE: Surrogate threshold effect
